# Inducible T-Cell Costimulator Mediates Lymphocyte/Macrophage Interactions During Liver Repair

**DOI:** 10.3389/fimmu.2021.786680

**Published:** 2021-12-03

**Authors:** Naresh Naik Ramavath, Laila Lavanya Gadipudi, Alessia Provera, Luca C. Gigliotti, Elena Boggio, Cristina Bozzola, Emanuele Albano, Umberto Dianzani, Salvatore Sutti

**Affiliations:** Department of Health Sciences and Interdisciplinary Research Centre for Autoimmune Diseases, University of East Piedmont, Novara, Italy

**Keywords:** acute liver injury, liver inflammation, liver healing, carbon tetrachloride poisoning, macrophage phenotype

## Abstract

The liver capacity to recover from acute liver injury is a critical factor in the development of acute liver failure (ALF) caused by viral infections, ischemia/reperfusion or drug toxicity. Liver healing requires the switching of pro-inflammatory monocyte-derived macrophages(MoMFs) to a reparative phenotype. However, the mechanisms involved are still incompletely characterized. In this study we investigated the contribution of T-lymphocyte/macrophage interaction through the co-stimulatory molecule Inducible T-cell co-stimulator (ICOS; CD278) and its ligand (ICOSL; CD275) in modulating liver repair. The role of ICOS/ICOSL dyad was investigated during the recovery from acute liver damage induced by a single dose of carbon tetrachloride (CCl_4_). Flow cytometry of non-parenchymal liver cells obtained from CCl_4_-treated wild-type mice revealed that the recovery from acute liver injury associated with a specific up-regulation of ICOS in CD8^+^ T-lymphocytes and with an increase in ICOSL expression involving CD11b^high^/F4-80^+^ hepatic MoMFs. Although ICOS deficiency did not influence the severity of liver damage and the evolution of inflammation, CCl_4_-treated ICOS knockout *(ICOS^-/-^*) mice showed delayed clearance of liver necrosis and increased mortality. These animals were also characterized by a significant reduction of hepatic reparative MoMFs due to an increased rate of cell apoptosis. An impaired liver healing and loss of reparative MoMFs was similarly evident in ICOSL-deficient mice or following CD8^+^ T-cells ablation in wild-type mice. The loss of reparative MoMFs was prevented by supplementing CCl_4_-treated *ICOS^-/-^* mice with recombinant ICOS (ICOS-Fc) which also stimulated full recovery from liver injury. These data demonstrated that CD8^+^ T-lymphocytes play a key role in supporting the survival of reparative MoMFs during liver healing trough ICOS/ICOSL-mediated signaling. These observations open the possibility of targeting ICOS/ICOSL dyad as a novel tool for promoting efficient healing following acute liver injury.

## Introduction

Acute liver injury resulting from viral infections, ischemia/reperfusion or adverse drug responses is the main factor in the pathogenesis of acute liver failure (ALF), a syndrome characterized by high mortality in the absence of immediate intensive care and/or emergency liver transplantation (1, 2). It is increasingly clear that beside hepatocyte damage inflammatory reactions play an important role in the pathogenesis of acute liver injury and are critical in the processes of parenchymal regeneration (3, 4). Animal studies have shown that inflammatory responses triggered by hepatocyte damage largely depend upon the massive recruitment and activation of granulocytes and monocytes-derived macrophages (MoMFs), which, on their turn, contribute to tissue damage by releasing pro-inflammatory mediators, reactive oxygen species (ROS), nitrogen monoxide (NO) and granule enzymes (3, 4). Consistently, interfering with granulocyte and MoMF recruitment has been shown to ameliorate liver injury (5, 6). Nonetheless, upon cessation of parenchymal damage MoMFs undergo functional changes characterized by the downregulation of the pro-inflammatory activity and the stimulation in the capability of scavenging death cells and of promoting tissue repair (7, 8). Furthermore, MoMFs contribute to replacing Kupffer cells lost during acute liver injury (9). This phenotype switch associates with the down modulation of the monocyte marker lymphocyte antigen 6 (Ly6C), also known as tissue plasminogen activator receptor, and of CCL2 chemokine receptor CCR2 with the concomitant increase in the expression of fractalkine receptor (CX_3_CR1) (10, 11) as well as of the mannose receptor (CD206) and the efferocytosis receptor c-Met Proto-Oncogene Tyrosine Kinase (MerTK) (9). Modifications in the cytokine milieu, efferocytosis of apoptotic bodies as well as the action of interleukin 4 (IL-4) are involved in promoting MoMF switching to a pro-repair phenotype (9). However, it is likely that additional cell-mediated signals might be also involved in modulating MoMF functions, since it is now evident that direct intracellular interactions in liver niche environment are critical for MoMF differentiation to Kupffer cells (12).

Previous studies have shown that lymphocyte interaction with myeloid cells mediated by Inducible T-cell costimulator (ICOS; CD278) and its ligand (ICOSL; CD275, also named B7h, B7-H2) potentiates lymphocyte IL-4 production (13) and contributes to skin wound healing in mice (14). ICOS belongs to the CD28 family of co-stimulatory molecules, and it is selectively expressed by activated T-cells, while its ligand is constitutively present on the surface of a variety of cells including dendritic cells, macrophages, B-cells but also on endothelial cells, lung epithelial cells and fibroblasts (13). The triggering of ICOS on T-cells by ICOSL has been shown to modulate lymphocyte cytokine secretion pattern and, in some conditions, to favour regulatory T- cell (Treg) differentiation (15). In addition, ICOS/ICOSL interaction plays an important role in the development and differentiation of Follicular T-helper cells (Tfh) in the germinal centres of lymphatic nodes (13). However, recent reports have shown that ICOS/ICOSL interaction can also trigger reverse signals able to modulate the functions of ICOSL-expressing cell. For instance, ICOSL-mediated signals favour dendritic cells maturation stimulating cytokine secretion and antigen presentation (16, 17), while they prevent monocytes differentiation to osteoclasts (18).

From this background, here, we investigated the contribution of ICOS/ICOSL dyad in modulating lymphocytes/MoMFs interactions in the evolution of acute liver damage.

## Materials and Methods

### Mice and Experimental Protocol

ICOS deficient (*ICOS^-/-^*; strain B6.129P2-Icos^tm1Mak^/J) and ICOSL deficient (*ICOSL^-/-^*; B6.129P2-Icosl^tm1Mak^/J) mice in C57BL/6 background were obtained from The Jackson Laboratories (Bar Harbor, Maine, USA). C57BL/6 wild-type (WT), *ICOS^-/-^* and *ICOSL^-/-^* mice were housed in pathogen-free conditions and fed *ad libitum* with standard chow diet and water. Liver injury was induced by injecting intra-peritoneally eight-week-old male mice with carbon tetrachloride (CCl^4^) (0.6 ml/kg in olive oil). Control animals received an injection with olive oil alone. Murine recombinant ICOS bound to the human IgG1 Fc portion (ICOS-Fc) was prepared as previously described (17). ICOS-Fc (100 µg in sterile saline) was administered to mice by intraperitoneal injection 24 hours after CCl_4_ and then every 12 hours up to 48 hours. Control animals received a similar amount of saline alone. Hepatic CD8^+^ T cell ablation was obtained by injecting WT mice with rat IgG2b anti-mouse CD8β monoclonal antibody (clone YTS 156.7.7). The animals received YTS 156.7.7 mAb (100 µg) three days before and immediately after CCl_4_ challenge. Control animals received the same amount of isotype matched IgGs. YTS 156.7.7 anti-CD8β monoclonal antibody was a kind gift by Dr. Stephen Cobbold, William Dunn School of Pathology, University of Oxford, (Oxford, UK). All animals were euthanized 24-72 hours after CCl_4_ administration.

### Assessment of Liver Injury

Livers were rapidly removed, rinsed in ice-cold saline, and cut into five pieces. Aliquots were immediately frozen in liquid nitrogen and kept at −80°C until analysis. Two portions of the left lobe from each liver were fixed in 10% formalin for 24h and embedded in paraffin. 4 µM tick liver sections were stained with hematoxylin/eosin using a Roche Ventana HE 600 automatic staining system (Roche Diagnostics International AG, Rotkreuz, Switzerland) and microphotographs were taken using a Nikon Eclips CI microscope fitted with a DSR12 camera (Nikon Europe BV, Amsterdam, Netherlands) using the NIS-Elements F4.60.00 acquisition software. The extension of necrotic areas was assessed morphologically in hematoxylin/eosin-stained liver sections using the ImageJ 1.53e software (National Institute of Health, Bethesda, MD, USA). The immunostaining for the proliferation marker Ki67 and ICOSL was performed in paraffin fixed section using anti-Ki67 monoclonal antibody (clone 30-9) and anti-ICOSL goat polyclonal antibodies (CD275 cod PA5-47161) (Thermo Fisher Scientific, Milano, Italy) in an automated staining system (BenchMark ULTRA IHC/ISH System, Roche Diagnostics International AG, Rotkreuz, Switzerland). In some experiments the staining with anti-ICOSL antibodies was also evidenced in frozen liver sections by immunofluorescence using Alexafluor^®^-546 donkey anti-goat IgG (H+L) (Thermo Fisher Scientific, Milano, Italy) and Leica DM 5500B fluorescence microscope and Leica Application Suite X (Leica Microsystems (Buccinasco, Italy). Plasma ALT levels were determined by a spectrometric kit supplied by Gesan Production SRL (Campobello di Mazara, Italy).

### Flow Cytometry Analysis of Liver Leukocytes

Livers were digested by type IV collagenase from Clostridium histolyticum (Sigma-Aldrich, St. Louis, MO, USA), and intrahepatic leukocytes were isolated by multiple differential centrifugation steps according to (19). The cell preparations were then subjected to red cell lysis by RBC Lysis Buffer (eBioscience, Thermo Fisher Scientific, Milano, Italy) and stained using combinations of the following monoclonal antibodies: CD45 (Clone 30-F11, Cat. 12-0451-82), CD3 (Clone 17A2, Cat. 17-0032-82) CD4 (Clone GK1.5, Cat. N. 56-0041-80) CD8 (Clone 53-6.7, Cat. 11-0081-82), Ly6C (Clone HK1.4, Cat. 53-59-32-80), NK1.1 (Clone PK136, Cat. 12-5941-81), Ly6G (Clone RB6-8C5, Cat. 47-5931-82), MHCII (Clone M5/114.15.2, Cat. 56-5321-80), CD103 (Clone 2E7, Cat. 12-1031-81), CD69 (Clone H1.2F3, Cat. 12-0691-82), CD107a (Clone eBio1D4B, Cat. N. 12-1071-81), CD206 (Clone MR6F3, Cat. 25-2061-80) MerTK (Clone DS5MMER, Cat. N. 56-5751-80 eBioscience, Thermo Fisher Scientific, Milano, Italy), CD11b (Clone M1/70, Cat. 101212), ICOS (Clone 15F9, Cat. 107705), ICOSL (Clone HK5.3, Cat. 107405), F4-80 (Clone BM8, Cat. 123113, Biolegend, San Diego, CA, USA), TREM-2 (Clone 78.18, Cat. MA5-28223, Thermo Fisher Scientific, Milano, Italy). In some experiments, macrophage viability was evaluated by cell staining using the rh-annexin V/FITC kit (Bender Med Systems, Vienna, Austria). Sample analysis was performed using the Attune NxT flow-cytometer (Thermo Fischer Scientific, Waltham, MA, USA) and data were elaborated with FlowJo™ Software (BD Biosciences, San Jose, CA, USA).

### mRNA Extraction and Real-Time PCR

mRNA was extracted from snap-frozen liver fragments using the TRIzol^®^ Reagent (Thermo Fischer Scientific, Milano, Italy) as previously reported (20). cDNA was generated from 1 µg of mRNA using the High-Capacity cDNA Reverse Transcription Kit (Applied Biosystems Italia, Monza, Italy) in a Techne TC-312 thermocycler (TecneInc, Burlington NJ, USA). Real-Time PCR was performed in a CFX96™ Real-time PCR System (Bio-Rad, Hercules, California, USA) using TaqMan Gene Expression Master Mix and TaqMan Gene Expression probes for mouse TNF-α (Mm99999068_m1), CCL2 (Mm00441242_m1), CD11b (Mm00434455_m1), TREM-1 (Mm01278455_m1), CD206 (Mm01329362_m1), TREM-2 (Mm04209422_m1), MerTK (Mm00434920_m1), CX_3_CR1 (Mm00438354_m1) and beta-actin (Cat. N 4352663, Applied Biosystems Italia, Monza, Italy). All samples were run in duplicate, and the relative gene expression was calculated as 2^-ΔCt^ over that of β-actin gene.

### Data Analysis and Statistical Calculations

Statistical analyses were performed by SPSS statistical software (SPSS Inc. Chicago IL, USA) using one-way ANOVA test with Tukey’s correction for multiple comparisons or Kruskal-Wallis test for non-parametric values. Significance was taken at the 5% level. Normality distribution was assessed by the Kolmogorov-Smirnov algorithm.

## Results

### ICOS Is Up Regulated in T-Lymphocytes in Response to Acute Hepatic Injury

**Figure 1:**
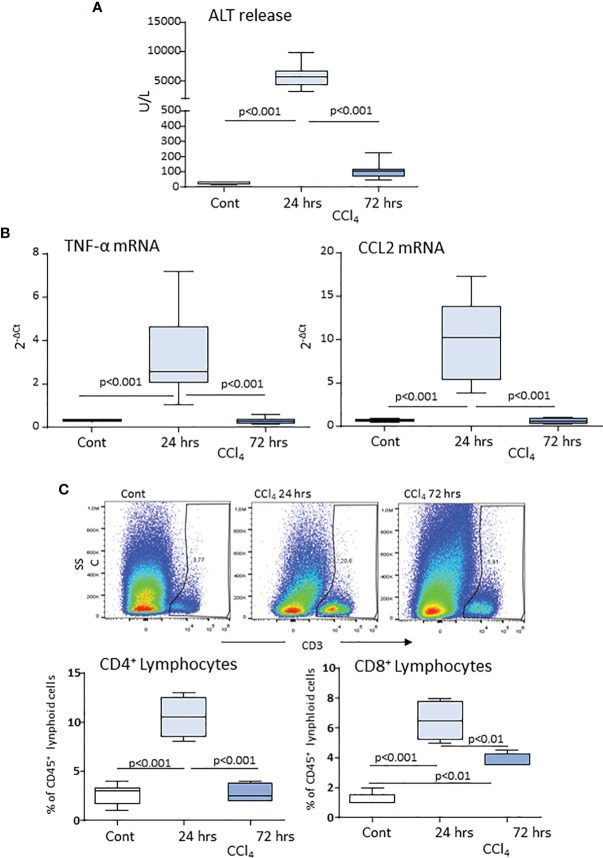
Hepatic lobular inflammation following acute liver injury promotes the liver recruitment of lymphocytes. Parenchymal damage, lobular inflammation and lymphocyte distribution in response to acute liver injury were analyzed in wild-type mice 24 and 72 hours after receiving an acute dose of CCl_4_. Circulating levels of alanine aminotransferase (ALT) **(A)**. Real-Time PCR analysis of the hepatic expression of the macrophage inflammatory markers TNF-α and CCL2 **(B)**. Flow cytometry analysis of the liver distribution of CD45^+^/CD3^+^/CD4^+^ helper and CD45^+^/CD3^+^/CD8^+^ cytotoxic T cells, **(C)**. The values are expressed as mean ± SD of 5-6 animals.

**Figure 2:**
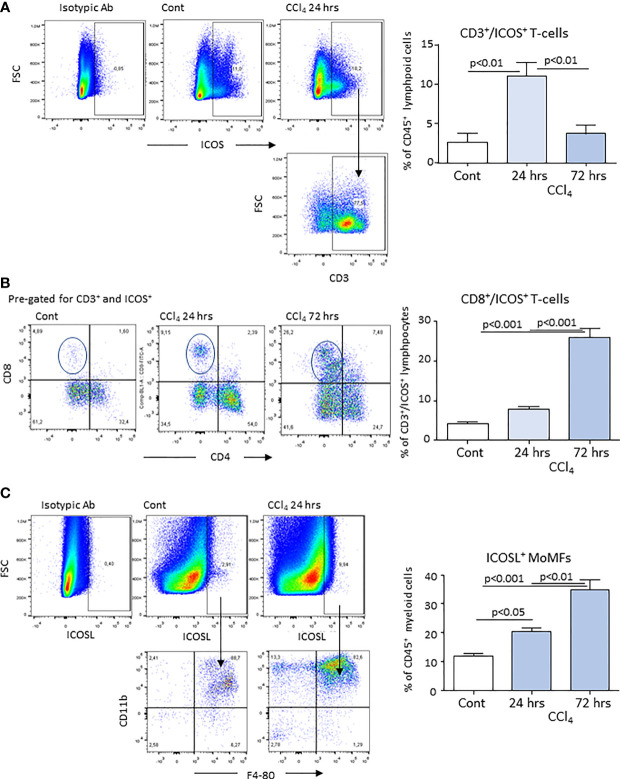
Expression of ICOS and ICOSL by lymphocytes and monocyte-derived macrophages (MoMFs) infiltrating the liver during acute inflammation. Liver inflammation was induced in response to acute hepatocyte injury caused by carbon tetrachloride (CCl_4_) in wild-type mice. The expression of ICOS was evaluated by flow cytometry in CD3^+^ T-lymphocytes infiltrating the liver of control (Cont) and CCl_4_-treated mice following 24 or 72 hours after challenge with the toxic agent **(A)**. Characterization of ICOS-expressing CD3^+^/CD4^+^ helper and CD3^+^/CD8^+^ cytotoxic T-cells in the liver of mice receiving CCl_4_ for 72 hours **(B)**. Flow cytometry analysis of ICOSL expression among CD11b^+^/F4-80^+^ MoMFs **(C)**. The values are expressed as mean ± SD of 3-4 different cell preparations.

According to previous studies (20, 21), acute liver injury caused by mice poisoning with the hepatotoxic agent carbon tetrachloride (CCl_4_) leads to centrilobular necrosis and an extensive inflammatory response which peaked after about 24 hours and rapidly declined thereafter with an almost complete recovery after 72 hours (**Figures 1A, B**). Beside liver infiltration by phagocytes, hepatic inflammation also involved the recruitment of both CD4^+^ helper and CD8^+^ cytotoxic T-lymphocytes (**Figure 1C**). Flow cytometry analysis showed that liver infiltrating T-lymphocytes expressed ICOS and that the overall prevalence of CD3^+^/ICOS^+^ cells followed the evolution of the inflammatory process, being maximal around 24 hours and declining in the recovery phase, mainly in relation to the lowering in CD4^+^ T-cell prevalence (**Figure 2A**). However, liver healing did not affect the prevalence of ICOS-positive CD8^+^ T-cells infiltrating the liver after 72 hours, which instead further increased as compared to early time points (**Figure 2B**). These CD8^+^ T lymphocytes comprised both CD103^+^ and CD103^-^ cells and expressed the activation markers CD69 and CD107a (**Supplementary Figure 1**). In the same animals, we also observed an early up regulation in the hepatic expression of ICOSL, which mainly involved CD11b^high^/F4-80^+^ MoMFs, (**Figure 2C**). The fraction of ICOSL-positive MoMFs was well appreciable at the peak of inflammation and further increased during the recovery phase (**Figure 2C**). Consistently, immunostaining with an anti-ICOSL polyclonal serum of liver sections from CCl_4_-treated at 72 hours showed selective labelling of infiltrating MoMFs in centrilobular areas, while no staining was appreciable in control livers (**Supplementary Figure 2**).

### ICOS Signaling Did Not Affect the Evolution of Inflammatory Responses Associated to Acute Liver Injury

From these data, we investigated whether interfering with ICOS/ICOSL signalling might affect the evolution of damage-associated hepatic inflammation. Time course analysis of the liver transcripts for pro-inflammatory markers in wild-type (WT) and ICOS knockout (*ICOS^-/-^*) mice receiving CCl_4_ showed the same pattern in the up-regulation and decline of tumor necrosis factor-α (TNF-α), CCL2 and the leukocyte integrin αM (CD11b) mRNAs, even though the down-modulation of these markers was more rapid in *ICOS^-/-^* mice (**Supplementary Figure 3**). In line with these findings, also the expression of the Triggering Receptor Expressed on Myeloid cells 1 (TREM-1), a membrane receptor involved in regulating the pro-inflammatory activity of neutrophils and macrophages (22, 23), was comparable in the two strains (**Supplementary Figure 2**). Consistently, flow cytometry analysis of liver MoMFs did not evidence appreciable difference in the hepatic recruitment of Ly6C^high^/CD11b^high^/F4-80^+^ pro-inflammatory MoMFs between WT and *ICOS^-/-^* mice 24 hours after CCl_4_ administration (**Supplementary Figure 4A**). ICOS absence also did not affect the functional maturation of MoMFs with the expression of high levels of Class II Mayor Histocompatibility Complex (MHCII) (**Supplementary Figure 3A**) as well as the up regulation of the fractalkine receptor CX_3_CR1 (**Supplementary Figure 4B**) (11, 21).

### ICOS Deficiency Interferes With Liver Healing Following Acute CCl_4_ Poisoning

**Figure 3:**
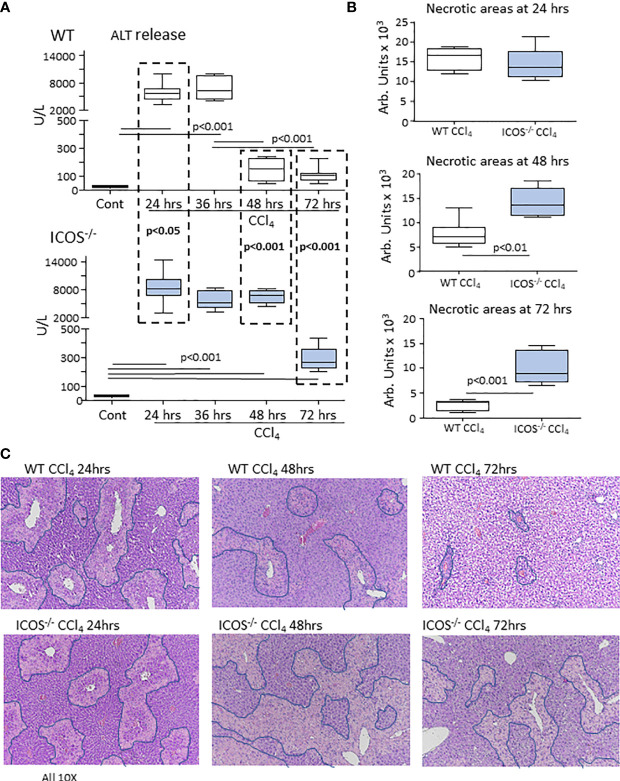
ICOS deficiency impairs the resolution of acute liver injury. The evolution of acute liver injury induced by the administration of CCl_4_ in wild-type and *ICOS^-/-^* mice was monitored by measuring alanine aminotransferase (ALT) release **(A)** and by morphometric evaluation of necrotic areas (encircled by blue lines) in liver sections stained with hematoxylin/eosin (magnification 10X) **(B, C)**. The results are expressed as mean ± SD of 5-8 animals for each time point.

In line with the pattern of liver inflammation, we observed that after 24 hours from CCl_4_ challenge, the extent of hepatic injury was similar in WT and *ICOS^-/-^* mice, despite circulating alanine aminotransferase (ALT) was slightly higher in these latter (**Figure 3A**). However, while in WT mice hepatic circulating ALT appreciably declined after 48 hours from CCl_4_ poisoning and liver damage almost completely recovered within 72 hours, ALT elevation persisted in *ICOS^-/-^* mice at 48 hours (**Figure 3A**). Liver histology confirmed that *ICOS^-/-^* mice failed to clear centrilobular necrosis during the recovery from CCl_4_ intoxication (**Figures 3B, C**). Furthermore, 5 out 13 (38%) of CCl_4_-treated *ICOS^-/-^* mice died by acute liver failure between 48 and 72 hours (**Supplementary Figure 5A**). At 72 hours the *ICOS^-/-^* mice who survived showed serum ALT about two folds higher than WT animals and still evident centrilobular necrotic areas (**Figures 3A–C**). In these animals, the immunostaining for the proliferation marker Ki67 did not evidence significant differences in the labelling of hepatocyte nuclei as compared to the wild-type littermates (**Supplementary Figure 5B**), suggesting that interfering with ICOS/ICOSL signaling does not affect hepatocyte regeneration but it might affect the responses of non-parenchymal cells.

### ICOS Signaling Is Required for the Reparative Response of Liver Macrophages

**Figure 4:**
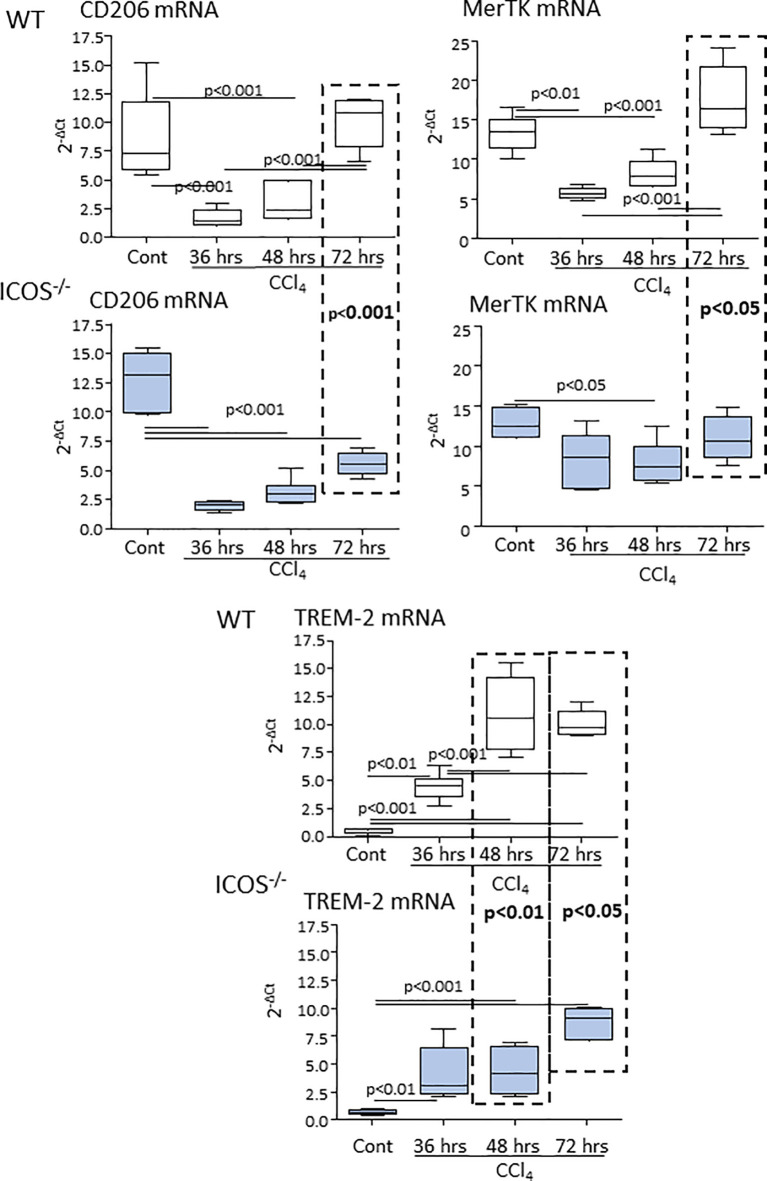
ICOS deficiency impairs the expression of reparative MoMF markers during the evolution of acute liver injury. The transcripts of reparative MoMF markers CD206, MerTK, and TREM-2 were evaluated by Real-Time PCR in the liver of wild-type and *ICOS^-/-^* mice at different time points following the administration of CCl_4_. The vertical dotted boxes indicate statistically significant differences between wild-type and *ICOS^-/-^* mice at each time point. The results are expressed as mean ± SD of 5-8 animals for each time point.

**Figure 5:**
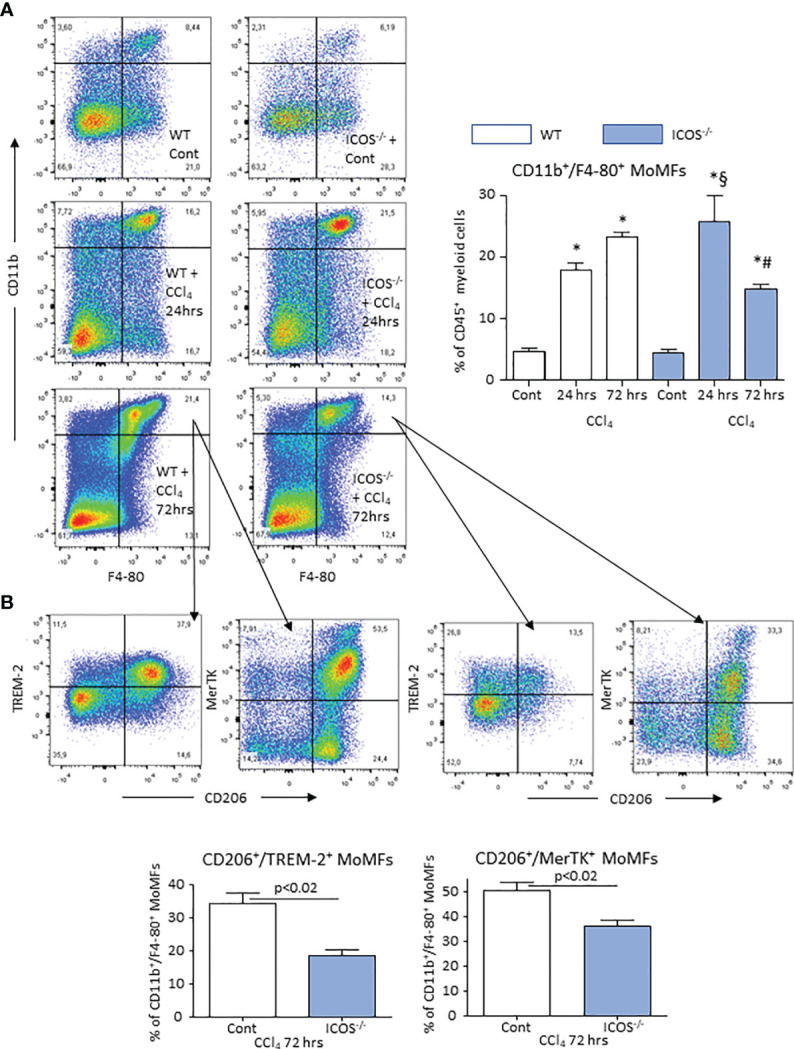
ICOS deficiency causes the loss of reparative MoMFs during the resolution of acute liver injury. The prevalence of liver CD11b^+^/F4-80^+^ MoMFs during the evolution of acute liver injury induced in wild-type and *ICOS^-/-^* mice receiving CCl_4_ was monitored by flow cytometry 24 or 72 hours after challenge with the toxic agent **(A)**. Characterization of the co-expression of CD206, TREM-2 and MerTK among CD11b^+^/F4-80^+^ MoMFs infiltrating the liver during the resolution of acute liver damage **(B)**. The results are expressed as mean ± SD of 3-4 different cell preparations. *p < 0.001 vs the relative controls; ^#^p < 0.01 vs WT CCl4 24 hrs; ^§^p < 0.01 vs WT CCl4 72 hrs.

From the observation that the absence of ICOS affected the recovery from acute hepatic injury, we investigated whether this might involve the switching of MoMF phenotype occurring during the healing response. The analysis of the transcripts for markers of reparative MoMFs such as the mannose receptor (CD206) and the efferocytosis receptor c-Met Proto-Oncogene Tyrosine Kinase (MerTK) (9), evidenced that the expression of both markers was dramatically reduced during acute inflammation and recovered to control values during liver repair (**Figure 4**). Similarly, hepatic healing was associated with the up-regulation in the Triggering Receptor Expressed on Myeloid cells 2 (TREM-2) (**Figure 4**), a plasma membrane receptor involved in the damping MoMF inflammatory response (24, 25). In these settings, *ICOS^-/-^* mice were less efficient than WT animals in up-regulating CD206, MerTK and TREM-2 (**Figure 4**). Furthermore, by evaluating the distribution of CD11b^+^/F4-80^+^ MoMFs 72 hours after CCl_4_ poisoning, we observed that the prevalence of MoMFs was lowered by about 30% in the livers of *ICOS^-/-^* mice (**Figure 5A**) and that such a loss involved the fraction of CD206/TREM-2/MerTK positive reparative MoMFs (**Figure 5B**). Along with the reduction in reparative MoMFs and the impaired clearance of necrotic areas, CCl_4_ challenged *ICOS^-/-^* mice also showed the persistence of a sustained hepatic infiltration by Ly6G^high^/CD11b^+^/F4-80^-^ granulocytes (3.2 ± 0.7% vs 8.6 ± 2.5% of CD45^+^ cells; p<0.01), while the prevalence of other myeloid cells was not affected.

### ICOS Expressing CD8^+^ T Cells Contribute to Liver Healing

**Figure 6:**
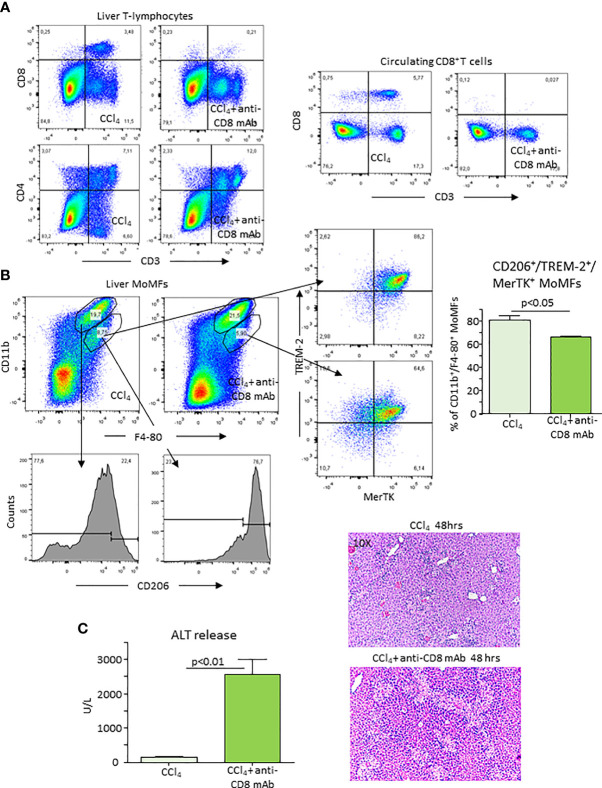
Depletion of CD8^+^ T-lymphocytes lowers reparative MoMFs and impairs the resolution of acute liver injury. Mice were treated with an anti-CD8 monoclonal antibody (mAb) 72 hours before and immediately after CCl_4_ administration and the evolution of hepatic injury was evaluated 48 hours after CCl_4_ poisoning. The distribution of liver and circulating CD4^+^ and CD8^+^ T cells and that of hepatic CD11b^+^/F4-80^+^ MoMFs was monitored by flow cytometry in mice receiving CCl_4_ **(A)**. The lowering of CD11b^+^/F4-80^+^ MoMFs induced by CD8 T-cell ablation involved CD206, TREM-2 and MerTK-expressing cells **(B)**. The effects of CD8^+^ T-depletion of the recovery from acute liver injury was evaluated by measuring alanine aminotransferase (ALT) release and histological analysis of liver sections stained with hematoxylin/eosin (magnification 10X) **(C)**.

As mentioned above, during the recovery from CCl_4_-induced hepatocyte injury, ICOS was prevalently expressed by liver CD8^+^ T cells, while ICOSL was up regulated in MoMFs. To explore the possibility that the interaction between CD8^+^/ICOS^+^ T cells and ICOS-L^+^ MoMFs might contribute to liver healing, we depleted liver CD8^+^ T cells using an anti-CD8 monoclonal antibody (mAb). **Figure 6A** shows that mice treatment with anti-CD8 mAb selectively lowered circulating and hepatic CD8^+^ T-cells. Furthermore, the overall prevalence of ICOS expressing CD3^+^ T cells within the liver was halved in the same animals 48 hours after CCl_4_ (3.8 ± 0.4% vs 2.2 ± 0.6% of CD45^+^ cells; p<0.02). CD8^+^ T-cell depletion also specifically reduced the fraction of CD11b^interm^/F4-80^+^/CD206^high^ cells that were TREM-2 and MerTK positive without affecting that of CD11b^high^/F4-80^+^/CD206^low^ MoMFs (**Figure 6B**). Such an effect was associated with a persistence of elevated ALT release and histological evidence of a delayed liver healing in CD8^+^ T cell-depleted mice 48 hours after CCl_4_ challenge (**Figure 6C**).

### ICOS-Mediated Signaling Is Required for the Survival of Liver Reparative Macrophages

**Figure 7:**
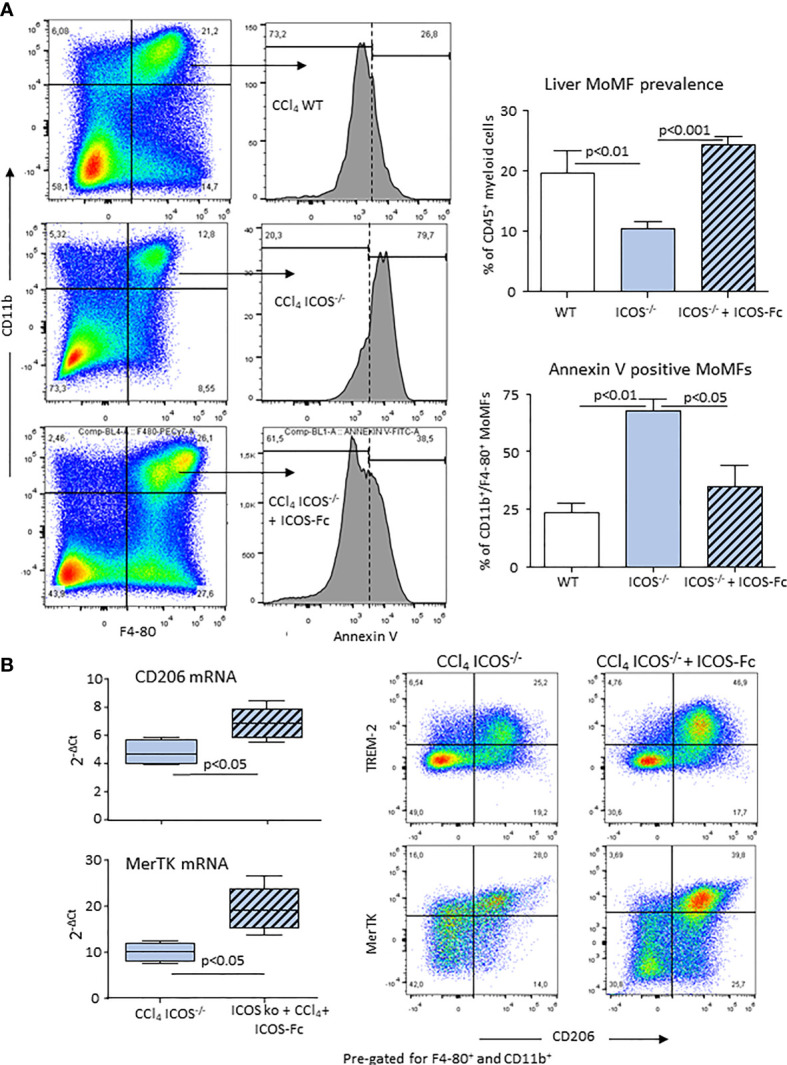
ICOS modulates the survival of reparative MOMFs during the resolution of acute liver injury. Annexin V staining of apoptotic CD11b^+^/F4-80^+^ MoMFs and the overall MoMF prevalence were evaluated by flow cytometry in the liver of wild-type and *ICOS^-/-^* mice 72 hours after receiving CCl_4_ in combination or not with recombinant ICOS-Fc treatment **(A)**. Real-Time PCR analysis of the hepatic transcripts for CD206 and MerTK and Hepatic distribution of CD206^+^/TREM-2^+^/MerTK^+^ reparative MoMFs in ICOS^-/-^ mice receiving CCl_4_ for 72 hours with or without ICOS-Fc supplementation **(B)**. The results are expressed as mean ± SD of 3-4 different cell preparations of 5-6 mice.

**Figure 8:**
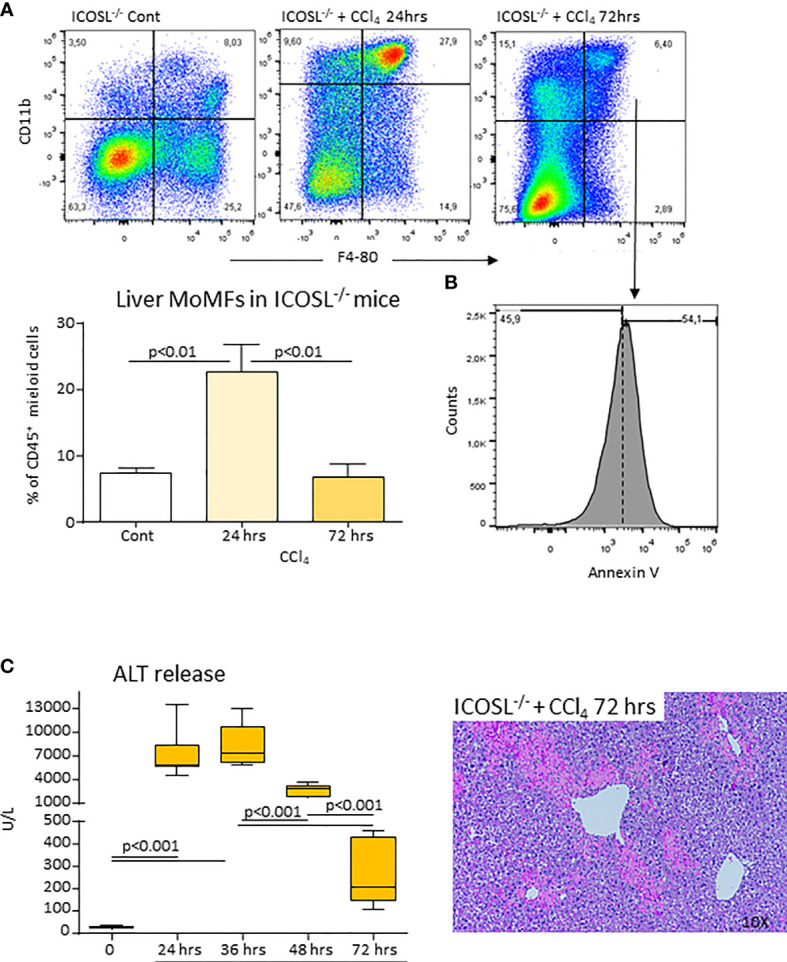
ICOSL deficiency of impairs MoMF survival and liver healing following acute liver injury. Wild-type and *ICOSL^-/-^* mice received CCl_4_ for 72 hours. The liver prevalence of MoMFs **(A)** and the expression of the apoptosis marker annexin V **(B)** were evaluated by flow cytometry. The evolution of liver damage was monitored by circulating alanine aminotransferase (ALT) and liver histology of liver sections stained with hematoxylin/eosin (magnification 10X) **(C)**. The results are expressed as mean ± SD of 3-5 animals for each time point.

From the above results and the observation that CD206^+^/TREM-2^+^/MerTK^+^ MoMFs expressed ICOSL (**Supplementary Figure 6**), we postulated that the reverse signaling triggered by the interaction of ICOS expressing CD8^+^ T-cells with ICOSL present in macrophages might influence the survival of reparative MoMFs. By labelling apoptotic cells with FITC-annexin V, we observed that the prevalence of annexin V-positive MoMFs in CCl_4_-poisoned *ICOS^-/-^* mice was more than two folds higher than in similarly treated wild-type littermates (**Figure 7A**). Interestingly, the treatment of *ICOS^-/-^* mice with murine recombinant ICOS bound to the human IgG Fc portion (ICOS-Fc) injected 24 hours after CCl_4_ completely prevented the loss of hepatic MoMFs and rescued them from apoptosis (**Figure 7A**). In particular, ICOS-Fc supplementation stimulated CD206 and MerTK transcripts and maintained the fraction of CD206^+^/TREM-2^+^/MerTK^+^ MoMFs (**Figure 7B**). The possible role of ICOSL-mediated reverse signaling in regulating the survival of reparative MoMFs was further supported by the observation that mice deficient for ICOSL (*ICOSL^-/-^*) showed a marked loss of MoMFs 72 hours after CCl_4_ poisoning (**Figure 8A**), which associated with an extensive annexin V staining (**Figure 8B**) and an impaired recovery from acute hepatic damage (**Figure 8C**).

### Recombinant ICOS Improves Acute Liver Damage in ICOS-Deficient Mice

**Figure 9:**
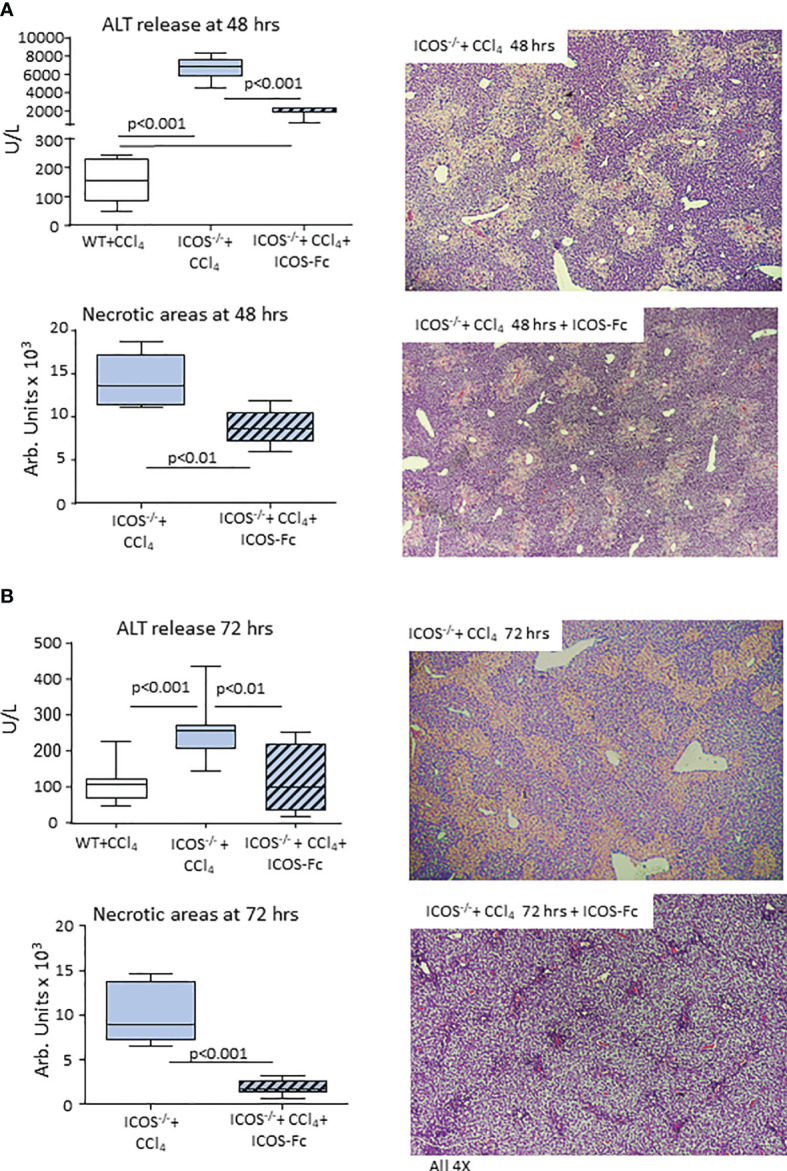
Mice supplementation with recombinant ICOS improves the recovery from acute liver injury. Wild-type and ICOS^-/-^ mice received CCl_4_ for 48 **(A)** or 72 hours **(B)** and the recovery from liver injury was evaluated by measuring alanine aminotransferase (ALT) release and by morphometric evaluation of necrotic areas in liver sections stained with hematoxylin/eosin (magnification 4X). The results are expressed as mean ± SD of 5-6 animals for each time point.

The observation that ICOS/ICOSL dyad was required for the survival of reparative liver MoMFs prompted us to evaluate the effects of ICOS-Fc on the evolution of CCl_4_-induced acute liver damage in *ICOS^-/-^* mice. Using the same protocol of ICOS-Fc administration effective in preventing MoMF apoptosis, we observed a significant improvement of ALT release and a reduction in the extension of necrotic areas 48 hours after CCl_4_ (**Figure 9A**). All *ICOS^-/-^* animals receiving ICOS-Fc (6 out of 6) survived CCl_4_ poisoning and histological evaluation revealed an almost complete recovery of liver necrosis after 72 hours (**Figure 9B**). Consistently, at this time point circulating ALT levels in *ICOS^-/-^* mice receiving ICOS-Fc were lower than in *ICOS^-/-^* mice receiving saline and comparable with those in WT mice (**Figure 9B**), supporting the hypothesis that ICOS-mediated signals are required for a full reparative response of liver infiltrating macrophages.

## Discussion

It is now well established that during the evolution of acute liver injury the phenotype switching of Ly6C^high^ pro-inflammatory MoMFs to Ly6C^low^ reparative MoMFs is critical for effective healing of parenchymal damage (9). Reparative MoMFs are, in fact, responsible for clearing death cells and cellular debris, remodeling extracellular matrix and producing cytokines and growth factors involved in promoting hepatocyte proliferation, such as IL-6, hepatocyte growth factor (HGF) and insulin-like growth factor (IGF) (9). Recent studies have shown that the reprogramming of liver MoMFs to the reparative phenotype involves IL-4 released by basophils and invariant natural killer T (iNKT) cells, as well as signals transduced by MerKT and TREM-2 receptors (9, 24, 25). Here, we show that beside these mechanisms, effective repair of acute liver damage also requires the presence of CD8^+^ T-lymphocytes expressing the co-stimulatory molecule ICOS. In fact, the deficit of ICOS, ICOS-L or CD8^+^ T-cells impairs the complete clearing of centrilobular necrosis 48-72 hours from acute liver damage induced by CCl_4_ poisoning. This effect is associated with an increase in parenchymal infiltration by granulocytes and an enhanced mortality of mice by acute liver failure.

Although the best characterized function of ICOS/ICOSL dyad is triggering ICOS on T-cells (13), growing evidence indicates that following the interaction with ICOS also ICOSL can transduce “reverse signals” in ICOSL expressing cells. ICOSL is constitutively present on antigen presenting cells (APCs) such as dendritic cells, macrophages and B-lymphocytes as well as on some non-lymphoid cells, such as endothelial cells and several tumour cells (13, 26, 27). In either human umbilical vein endothelial cells, dendritic cells and tumour cell lines ICOSL stimulation impairs the adhesive and migratory capacities (26, 27), while it modulates cytokine secretion and antigen presentation by monocyte-derived dendritic cells (16, 17). Furthermore, ICOSL signalling prevents the differentiation of monocyte to osteoclasts stimulated by receptor activator of NF-kB ligand (RANKL) (18). This latter effect involves the specific stimulation of p38 mitogen activated kinase (18), while the action in dendritic cell depends upon JNK and PKC transduction (17). Interestingly, ICOSL interaction with α_v_β_3_ integrin also modulates podocyte adhesion and ICOSL^-/-^ mice are more susceptible to proteinuria induced by endotoxin treatment or diabetic nephropathy (28).

We have shown that within the liver, ICOSL is mainly expressed by CD11b^high^/F4-80^+^ MoMFs recruited in response to parenchymal injury as well as by CD206^+^/MerKT^+^/TREM-2^+^ MoMFs associated with healing from acute damage. This is consistent with an early report showing that, differently from lung epithelial cells, hepatocytes did not express ICOSL (29). In line with the capacity of ICOSL-mediated signals of influencing monocyte-derived cells, we have observed that ICOS/ICOSL interaction is required for the survival of reparative MoMFs during the healing phase. In fact, both ICOS and ICOSL deficient mice show an enhanced rate of MoMF apoptosis 72 hours after CCl_4_, which can be rescued by the administration of soluble ICOS-Fc to ICOS^-/-^ mice. Mice treatment with ICOS-Fc also allows the full clearance of centrilobular necrosis. The involvement of MoMFs expressing MerKT and TREM-2 receptors in the recovery from acute liver injury is consistent with the notion that both MerKT or TREM-2 are required for damping macrophage pro-inflammatory functions and promoting the clearance of apoptotic cells (24, 25, 30). Moreover, recent reports have shown that mice deficient in either MerKT or TREM-2 suffer a delayed resolution of acetaminophen hepatotoxicity (24, 25, 30). Interestingly, the expansion of MerTK-positive MoMFs is also evident in the liver of patients with acute liver failure and associates with a greater severity of liver injury and adverse clinical outcome (30).

At present, the network of signals that promotes the survival of reparative MoMFs has not been investigated in detail. Previous studies have shown that early after infiltrating injured liver MoMFs up regulate the fractalkine receptor CX_3_CR1 as well as MerTK, both of which are involved in regulating their survival (21, 30). In our hands, the expression of CX_3_CR1 is unaffected in MoMFs from *ICOS^-/-^* mice, which instead show reduced MerTK transcripts. These data and the observation that reparative MoMFs are greatly lowered in the liver of MerTK-deficient mice during the recovery from acetaminophen poisoning (30) suggest the possibility that ICOS/ICOSL interaction might contribute to MerTK-mediated signals. Nonetheless, we cannot exclude that other receptors including TREM-2 might also be implicated, since TREM-2 is required for the survival of liver MoMFs and brain microglial cells (24, 31).

The involvement of ICOS/ICOSL dyad in liver repair is consistent with the report by Maeda and colleagues of a dramatic delay in skin wound healing in both *ICOS^-/-^* and *ICOSL^-/-^* mice (14). They observed that in both strains impaired wound healing associates with a strong reduction in keratinocyte migration, angiogenesis, and granulation tissue formation, as well as with a reduced wound infiltration by T-cells, macrophages, and neutrophils. These effects were mimicked by whole T-cell depletion, suggesting that signals involving ICOS were driving skin healing (14). However, in this setting, the supplementation with CD4^+^ T cells and IL-6 improved epidermis repair (14).

A further novel aspect emerging from this study regards the involvement of CD8^+^ T lymphocytes in the regulation of hepatic repair. So far, the role of lymphocytes in the evolution of acute liver injury has been incompletely characterized. The available evidence indicates that liver recruitment of B and T cells accompanies that of granulocytes and MoMFs following ischemia/reperfusion (I/R) or acetaminophen poisoning (32–34). However, while CD4^+^ T helper cells contributes to I/R liver damage likely by stimulating MoMFs *via* CD40/CD154 co-stimulatory molecules and interferon-γ secretion (34, 35), their role in other type of acute liver damage has not been investigated in detail. Here, we show that CD4^+^ and CD8^+^ T-cells are recruited within the liver in response to CCl_4_-induced hepatocyte injury and that CD8^+^ T-cells support the recovery phase by providing ICOS-mediated survival signals to reparative CD206^+^/MerKT^+^/TREM-2^+^ MoMFs. Previous studies have shown that regulatory T-cells (Tregs), promote repair and regeneration of various organs (36). Furthermore, Liew and co-workers have recently reported that iNKT cells secreting IL-4 are important in liver repair by stimulating MoMF phenotype switching (37). Our data are not in contrast to these observations but add further complexity to the mechanisms responsible for driving liver repair. Recent data indicate that IL-2-producing tissue resident/memory CD8^+^ T-cells are present within the liver where they patrol the vasculature and provide protection against invading pathogens (38). Thus, it is possible that, in response to an inflammatory environment, these cells up-regulate ICOS allowing them to stimulate ICOSL^+^ MoMFs. The involvement of direct cellular interaction in modulating liver MoMF functions is in line with a recent report by Sakai and co-workers (12) showing that the Notch and transforming growth factor-β family ligands produced by sinusoidal endothelial cells are required for inducing MoMF differentiation to Kupffer cells. However, further studies are required to better characterize the transcriptional changes associated with MoMF/CD8^+^ T-cell interaction within the liver also considering that single cell RNA-sequencing of cells obtained from livers of human cirrhotic patients has identified TREM-2/CD9 expressing MoMFs in the fibrotic niches (39).

In conclusion, our data demonstrated that MoMF interaction with CD8^+^ T-lymphocytes through the ICOS/ICOSL dyad plays an important role in liver healing after acute injury by supporting the survival of reparative MoMFs. These observations open the possibility of targeting ICOSL as a novel tool to promote healing responses following acute liver injury.

## Data Availability Statement

The raw data supporting the conclusions of this article will be made available by the authors, without undue reservation.

## Ethics Statement

The animal study was reviewed and approved by Italian Ministry of Health (authorization No. 84/2021-PR) according to the European legal requirements.

## Authors Contributions

NNR, LLG, and AP designed the study and performed the experiments. LCG and EB performed the experiments and analysed the data. CB analysed the data. EA, SS and UD contributed to the study design and supervised the study and the manuscript. SS and UD contributed to funding acquisition. All authors contributed to the article and approved the submitted version.

## Funding

This work was supported by the University of East Piedmont (grant FAR 2019) and Fondazione Cariplo Milan, Italy (grant 2017/0535) and Associazione Italiana Ricerca sul Cancro, Milan (grant IG20714). EB is supported by the Fondazione Veronesi, Milan, Italy.

## Conflict of Interest

UD, EB, and LCG are listed as inventors on the patent PCT/IB2019/050154 “Novel anti-tumor therapeutic agents”. EB, UD, and LCG are founders of an University Spin-off (NOVAICOS).

The remaining authors declare that the research was conducted in the absence of any commercial or financial relationships that could be construed as a potential conflict of interest.

## Publisher’s Note

All claims expressed in this article are solely those of the authors and do not necessarily represent those of their affiliated organizations, or those of the publisher, the editors and the reviewers. Any product that may be evaluated in this article, or claim that may be made by its manufacturer, is not guaranteed or endorsed by the publisher.

## Abbreviations

ALF, acute liver failure; CCl_4_ carbon tetrachloride; CX_3_CR1 fractalkine receptor; ICOS inducible costimulator; ICOS-L inducible costimulator ligand; MerTK c-Met Proto-Oncogene Tyrosine Kinase; MoMFs monocytes-derived macrophages; TREM-1 and 2 Triggering Receptor Expressed on Myeloid cells 1 and 2.
