# Supplementary material for: Inducible T-Cell Costimulator Mediates Lymphocyte/Macrophage Interactions During Liver Repair
Source: Front Immunol. 2021 Dec 3;12:786680. doi: 10.3389/fimmu.2021.786680 (PMC8678521; doi:10.3389/fimmu.2021.786680)
Supplement: Supplementary file 1 [file DataSheet_1.pdf]

Inducible Costimulatory Signal (ICOS) mediates lymphocyte/macrophage interactions during liver repair

Naresh Naik Ramavath*, Laila Lavanya Gadipudi*, Alessia Provera*, Luca C. Gigliotti, Elena Boggio, Cristina Bozzola, Emanuele Albano, Umberto Dianzani#, Salvatore Sutti#

Dept. of Health Sciences and Interdisciplinary Research Centre for Autoimmune Diseases, University of East Piedmont, Novara, Italy.

Supplementary materials

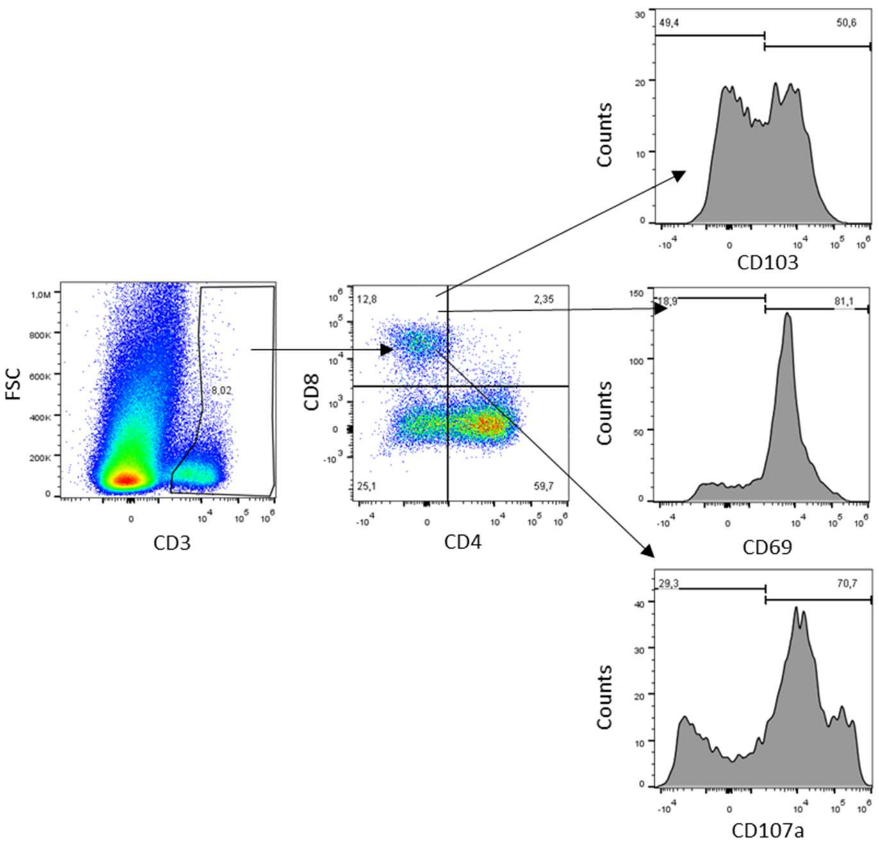

Supplementary Figure 1: Phenotypic characterization of ICOS expressing CD8+ T-lymphocytes.

The expression of CD103 and of the activation markers CD69 and CD107a was evaluated by flow cytometry in CD3+/CD8+ cytotoxic T-cells obtained from the livers of mice receiving CCl_4 for 72 hours.

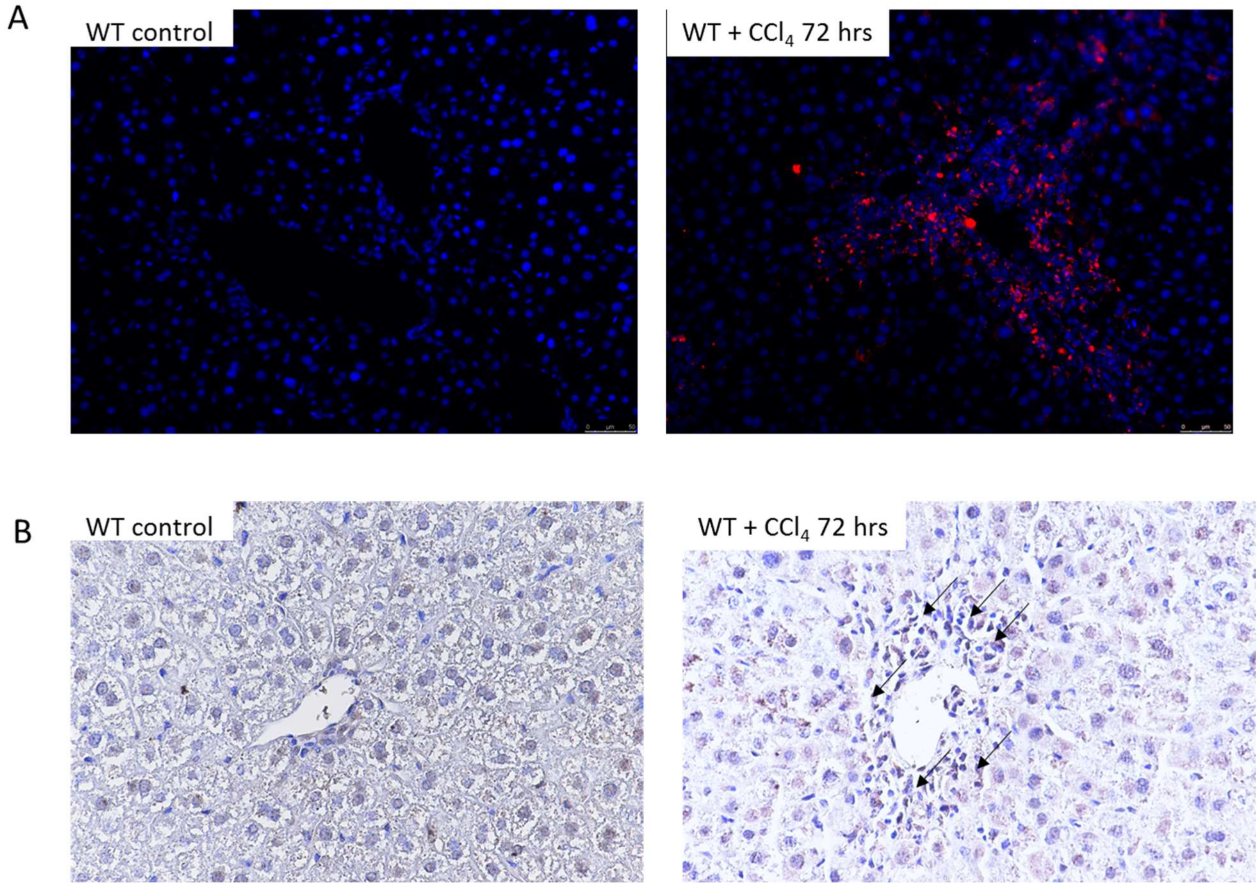

Supplementary Figure 2: ICOSL is specifically expressed by liver infiltrating MoMFs.

Wild-type mice received or not CCl_4 for 72 hours. MoMF staining with an anti-ICOSL polyclonal goat antibodies was evidenced by immunofluorescence (Panel A) in frozen liver sections using an Alexafluor®-labelled secondary antibody and confirmed by immunohistochemistry (Panel B) in paraffin fixed liver sections. Magnification 40X.

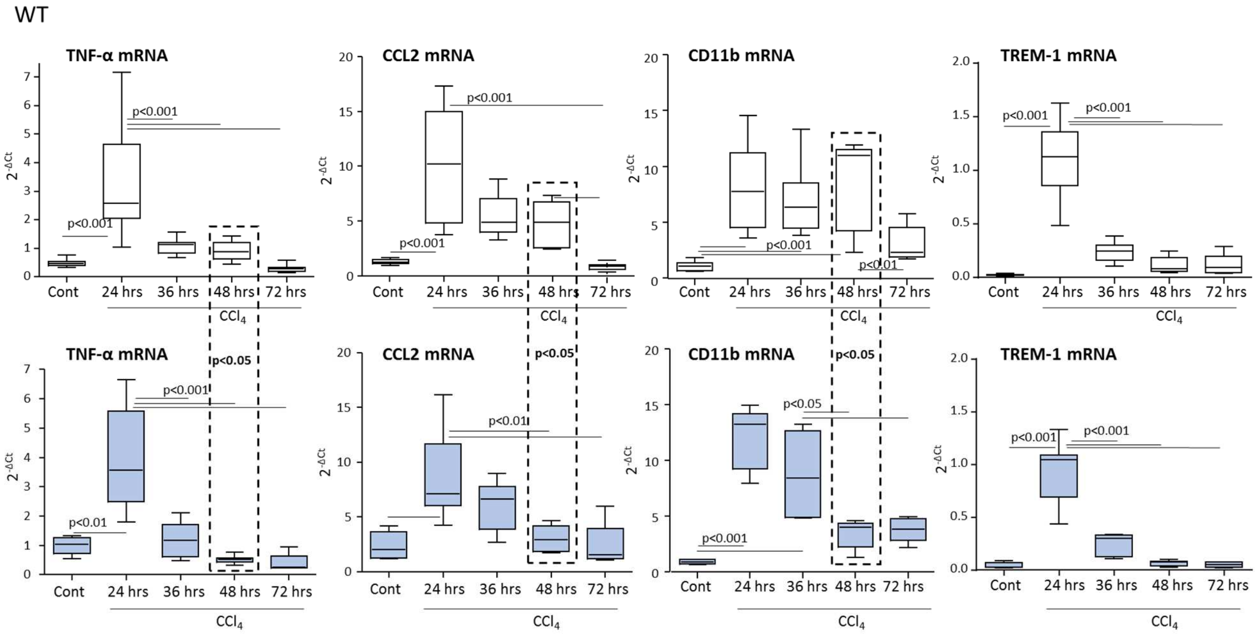

Supplementary Figure 3: ICOS deficiency does not interfere with the evolution of hepatic inflammation associated to acute liver injury.

The transcripts of the pro-inflammatory markers TNF-α, CCL2, CD11b and TREM-1 were evaluated by Real-Time PCR in the liver of wild-type and ICOS$^{-/-}$ mice at different time points following the administration of CCl_4. The results are expressed as mean ± SD of 5-8 animals for each time point. The vertical boxes indicate statistically significant differences between wild-type and ICOS$^{-/-}$ mice at each time point.

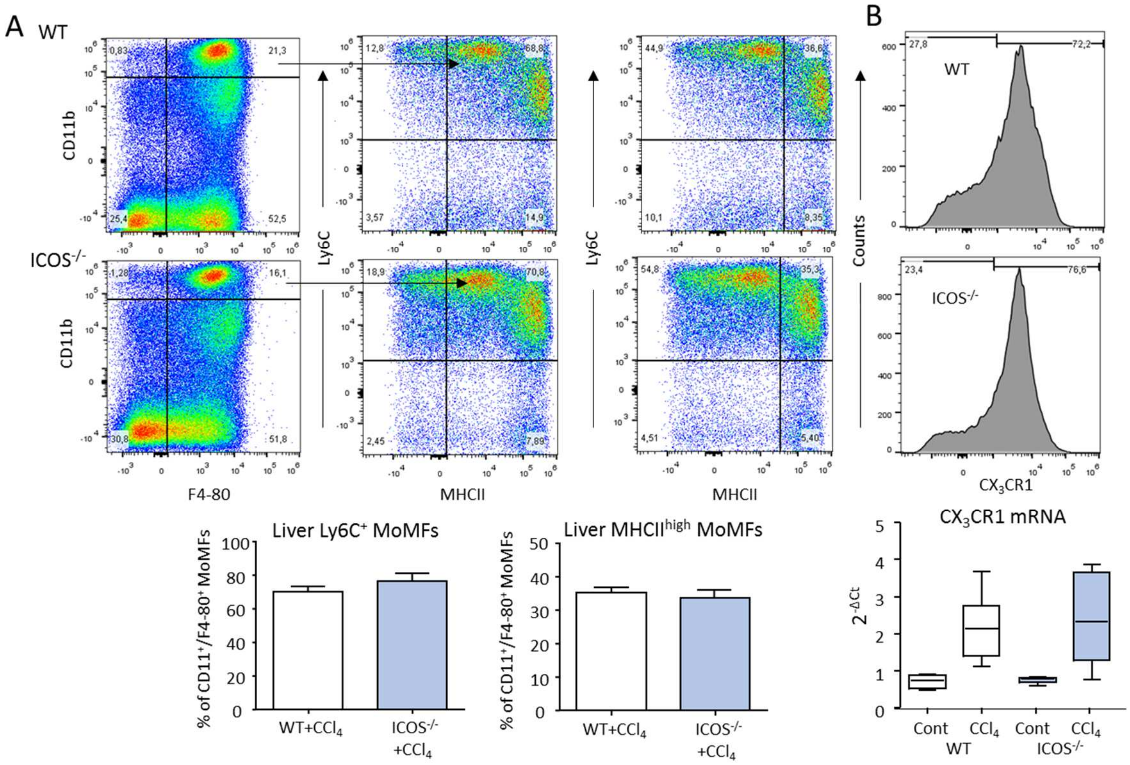

Supplementary Figure 4: ICOS deficiency does not interfere with the recruitment and maturation of inflammatory MoMFs in response to acute liver injury.

Wild-type and ICOS$^{-/-}$ mice received CCl_4 for 24 hours. (Panel A) The expression of Ly6C^{high} and MHCII was monitored by flow cytometry in liver CD11b^{high}/F4-80^{+} MoMFs. (Panel B) The expression of CX_3CR1 was evaluated by Real-Time PCR as well as by MoMF flow cytometry in the liver of wild-type (WT) and ICOS$^{-/-}$ mice 24 hours following the administration of CCl_4. The values are expressed as mean ± SD of three different cell preparations or 5-6 animals.

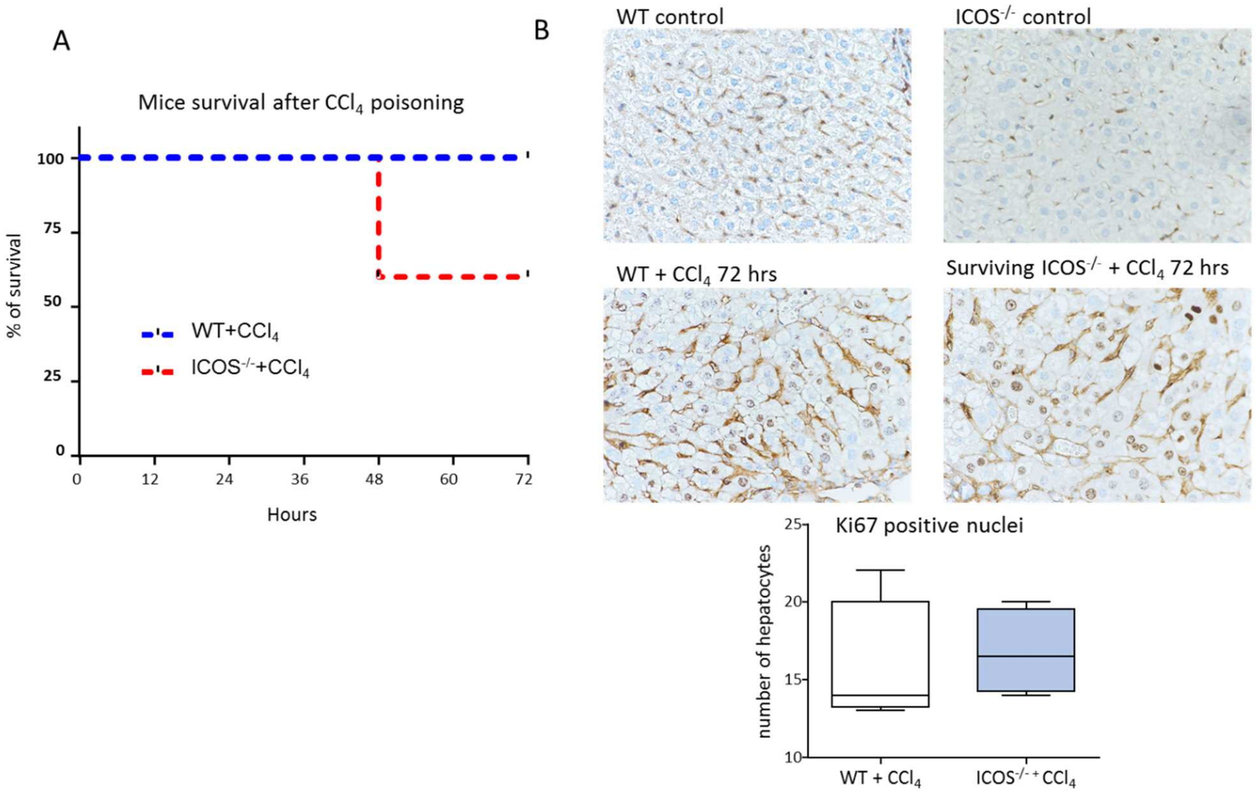

Supplementary Figure 5: ICOS deficiency enhances mice mortality following CCl_4 poisoning without affecting hepatocyte replicative capability.

Wild-type and $ICOS^{-/-}$ mice received CCl_4 for 24-72 hours. (Panel A) Kaplan-Mayer curve for hepatocyte survival following CCl_4 poisoning. (Panel B) Nuclear hepatocyte staining with the proliferation marker Ki67 was evaluated in paraffin fixed liver sections and the number of Ki67 positive nuclei was evaluated in five randomized areas. The results are expressed as percent of all nuclei and are means ± SD of 3-4 representative animals. Magnification 40X.

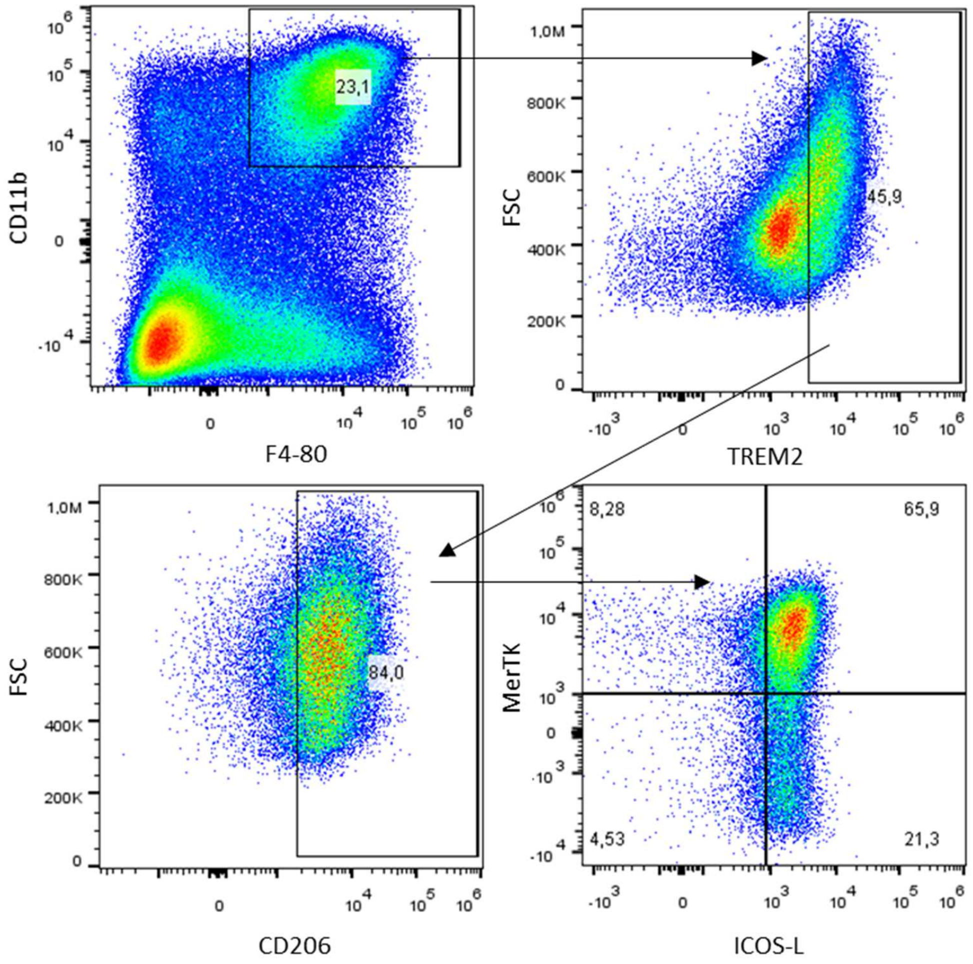

Supplementary Figure 6: Reparative MoMFs express ICOS-L.

The expression of ICOS-L was evaluated by flow cytometry in TREM-2$^+$, CD206$^+$, MerTK$^+$ and reparative CD11b$^+$/F4-80$^+$ MoMFs isolated from the liver of a wild-type mice.
